# Impact of controlled type 2 diabetes on muscle-tendon mechanics

**DOI:** 10.1007/s00592-026-02705-5

**Published:** 2026-05-06

**Authors:** Riccardo Magris, Andrea Monte, Nicolò Vigolo, Francesca Nardello, Michele Trinchi, Carlo Negri, Paolo Gisondi, Chiara Cosma, Giovanni Sartore, Annunziata Lapolla, Paolo Moghetti, Paola Zamparo

**Affiliations:** 1 Department of Neurosciences, Biomedicine and Movement Sciences, University of Verona, via Casorati 43, Verona, 37131 Italy; 2 Unit of Endocrinology, Diabetes and Metabolism, Department of Medicine, University of Verona, Verona, Italy; 3 Section of Dermatology and Venereology, Department of Medicine, University of Verona, Verona, Italy; 4 Department of Medicine, University of Padova, Padova, Italy

**Keywords:** Advanced glycation end products, Glycated haemoglobin, Tendon stiffness, Muscle stiffness

## Abstract

**Aims:**

This study aimed to investigate the impact of type 2 diabetes (T2D) on muscle and tendon mechanics by comparing individuals with controlled diabetes to a healthy cohort matched for age, BMI, and physical activity level. A secondary aim was to investigate the possible association between muscle-tendon proprieties and glycated haemoglobin (HbA1c) or advanced glycated end products (AGE, RAGE) as determined in blood and skin biopsies.

**Methods:**

Twenty-eight patients and eighteen controls were recruited for this study. Achilles tendon stiffness (k_T_), muscle-tendon stiffness (k_M_, in gastrocnemius medialis) and the rate of torque development (RTD) were evaluated by combining dynamometric and ultrasound data.

**Results:**

Diabetic patients showed increased tendon stiffness and reduced tendon elongation compared to controls, but similar RTD and k_M_ values. No differences in advanced glycation end products (in serum or biopsies) were observed between cohorts, but a significant positive correlation was observed between k_T_ and HbA1c (*r* = 0.610, *N* = 46, *P* < 0.001).

**Conclusion:**

Our data indicate that muscle, but not tendon, properties can be preserved in controlled and physically active diabetic patients and that higher tendon stiffness does not result in a functional deficit (i.e., same explosive capacity between cohorts). Although this study is cross-sectional and has a limited sample size, our data suggest a potential role of HbA1c as a non-invasive biomarker of altered tendon mechanics in people with diabetes.

**ClinicalTrials.gov, protocol number:**

NCT05585502.

**Supplementary Information:**

The online version contains supplementary material available at 10.1007/s00592-026-02705-5.

## Introduction

Type 2 diabetes (T2D) affects several tissues, apparatuses and systems [1–3]; so far, little attention has been paid to alterations in the musculoskeletal system, which may contribute to the decline of the general state of health of diabetic people [4] and may limit the therapeutic use of exercise in these subjects.

In this regard, T2D was associated with complications affecting both tendons and skeletal muscles, leading to reduced mobility, pain, and an increased healthcare burden. Tendon complications are particularly common in individuals with T2D, who face nearly four times the risk of developing Achilles tendinopathy compared to non-diabetic individuals [5]. Another complication is diabetic myopathy, which can lead to pain and swelling of the affected muscles, reducing locomotor functions [6]. Even if these pathologies are typically associated with poorly controlled diabetes [5], two recent studies [7, 8] observed impairment in tendon mechanical properties or joint cartilage quality (both assessed by MRI), even in individuals with early alterations in HbA1c levels, as seen in prediabetes.

From a mechanical point of view, Achilles tendon stiffness (e.g. a reduced tendon elastic capacity) was proved to be greater in diabetic patients compared to age-matched controls, the more so in those affected by peripheral neuropathies (e.g. [9, 10]). ; on the other hand, no studies investigated so far eventual structural and functional alterations in skeletal muscle (e.g. muscle stiffness) in diabetic patients.

Changes in Achilles tendon (and possibly plantar flexor) mechanical properties in people with T2D could affect the muscle’s mechanical output and daily life activities. For instance, changes in Achilles tendon and muscle stiffness could affect an individual’s explosive force capacity, reflecting the capacity to exert force rapidly in response to different tasks, including daily situations such as avoiding an obstacle while walking or recovering from an unexpected loss of balance [11].

The morphological abnormalities in the Achilles tendon of people with diabetes were suggested to result from nonenzymatic glycation of the collagen fibres [12]. However, more recent studies of human tendons reported no evidence of increased advanced glycation end-products (AGEs) cross-linking in the tendons of patients with diabetes [10, 13].

In light of this reasoning, the primary aim of this study was to investigate the impact of type 2 diabetes on muscle and tendon mechanics by comparing individuals with controlled diabetes to a healthy cohort matched for age, BMI, and physical activity levels.

The specific purposes were: *(i)* to characterise muscle and tendon function (e.g. rate of force development, maximal force capacity, muscle stiffness and tendon stiffness) in these cohorts; *(ii)* to test whether eventual muscle/tendon mechanical alterations are associated with increased levels of non-enzymatic glycation products (HbA1c, AGE or RAGE) in blood or skin biopsies.

## Materials and methods

### Participants

Twenty-eight T2D patients and eighteen healthy controls participated in this study (see Table 1 for participants’ characteristics and supplementary materials for sample size calculations). For all participants, inclusion criteria consisted of a body mass index between 23 and 30 kg m^− 2^, a moderate level of physical activity in everyday life, an age range of 50–70 years and no previous history of musculoskeletal injury of the lower limbs. For patients, exclusion criteria included: neuropathy of nondiabetic or diabetic origin; severe neuropathy; foot ulcers; arterial insufficiency; ankle/foot arthritis; previous foot/knee surgery; previous Achilles tendon rupture; previous Charcot foot; cardiovascular and respiratory deficits; insulin therapy.

**Table 1:** Participants’ characteristics (data are means ± standard deviation)

*n* (M/F)	T2D	CR
	28 (19/9)	18 (10/8)
Age (years)	62.5 ± 4.5	63.9 ± 5.3
Body mass (kg)	79.8 ± 10.8	73.0 ± 11.9
Stature (m)	1.72 ± 0.08	1.68 ± 0.09
BMI (kg m^− 2^)	26.9 ± 2.5	25.6 ± 2.8
Disease onset (years)	8.2 ± 7.2	-
HbA1c 3y (mmol molHb^− 1^)	50.7 ± 6.4	
HbA1c (mmol molHb^− 1^)	52.4 ± 8.8	36.4 ± 2.5**
HbA1c (%)	6.97 ± 0.84	5.48 ± 0.25**
Glucose (mmol L^− 1^)	7.18 ± 1.61	5.07 ± 0.44**
Creatinine (µmol L^− 1^)	77.5 ± 17.3	76.2 ± 12.5
Total Cholesterol (mmol L^− 1^)	3.86 ± 0.73	5.27 ± 1.05**
HDL cholesterol (mmol L^− 1^)	1.26 ± 0.28	1.65 ± 0.43*
Triglycerides (mmol L^− 1^)	1.33 ± 0.42	1.09 ± 0.50
AGE serum (µg mL^− 1^)	0.24 ± 0.13	0.28 ± 0.14
AGE skin (µg mL^− 1^)	0.03 ± 0.01	0.04 ± 0.02
RAGE serum (ng mL^− 1^)	0.68 ± 0.16	0.70 ± 0.17
RAGE skin (ng mL^− 1^)	0.13 ± 0.07	0.11 ± 0.14
ADL (score)	8.0 ± 0.0	8.0 ± 0.0
IADL (score)	6.0 ± 0.0	6.0 ± 0.0
IPAQ (MET-min week^− 1^)	1158 ± 314	1597 ± 571*
MMSE (score)	28.9 ± 1.2	28.9 ± 1.1

*IADL* Instrumental Activities Daily Living, *ADL* Activities of Daily Living questionnaires, *IPAQ* International Physical Activity Questionnaire, *MMSE* Mini-Mental State Examination. Unpaired t-test: * *p* < 0.05; ** *p* < 0.001

All patients were referred to the outpatient clinic of the Division of Endocrinology, Diabetes and Metabolism of Verona City Hospital (Italy). The antidiabetic therapies for the patients were the following: diet only (14.2%), metformin (82.1%), sulphonylureas (10.7%), DPP4i (dipeptidyl peptidase 4 inhibitors, 14.3%), SGLT2i (sodium-glucose cotransporter-2-inhibitors, 25%), GLP-1a (glucagon-like peptide-1 receptor agonists, 25%).

The level of functional capacity was assessed using the IADL (Instrumental Activities Daily Living) and the ADL (Activities of Daily Living) questionnaires [14]; the level of physical activity was assessed using the IPAQ (International Physical Activity Questionnaire [15]), ; cognitive function was assessed using the Mini-Mental State Examination (MMSE [16]), .

The study conformed to the Declaration of Helsinki for the study on human subjects. The experimental protocol was approved by the local ethical committee (CESC AOUI, Verona; protocol number: 40428) and registered as a clinical trial (ClinicalTrials.gov, protocol number: NCT05585502). All participants provided their written informed consent for the experimental procedures.

### Experimental design

A preliminary session involved assessment of the inclusion/exclusion criteria, the questionnaire and a blood and skin sampling. In the following days, the participants were requested to perform: (i) maximal fixed-end voluntary contractions (MVC) to determine the medial gastrocnemius (GM) muscle-tendon stiffness (k_M_) and the Achilles tendon stiffness (k_T_) and (ii) maximal explosive fixed-end contractions to determine peak torque and the rate of torque development (RTD).

### Blood samples and biopsies

Blood samples of 15 ml were collected (according to the standard clinical management of patients with type 2 diabetes) to assay serum glucose, triglycerides, HDL cholesterol, total cholesterol, creatinine, and HbA1c. Serum was stored for subsequent AGE and RAGE quantification. In diabetic patients, average HbA1c over the past three-years was also obtained from their medical records.

Skin biopsies were then performed to determine AGE and RAGE content (see supplementary materials for details). Blood samples were obtained from all participants; four T2D patients and seven controls declined consent for the skin biopsies.

### Evaluation of muscle and tendon stiffness

The torque-elongation curves of the gastrocnemius medialis muscle belly and of the Achilles tendon were obtained by combining torque and ultrasound measurements during a series of maximal voluntary contractions (MVCs). Torque values were determined using a dynamometer (Cybex NORM, Lumex Inc., Ronkonkoma, New York, USA) while an ultrasound apparatus (Telemed Mycrus Ext-1, Lituania) was utilized to record the displacement of the muscle fibres and of the Achilles tendon.

For the muscle architecture measurements, a customised version of a semi-automatic tracking algorithm [17] was used to determine gastrocnemius medialis muscle thickness (MT) and pennation angle (PA). Fascicle length (FL) was then calculated using a trigonometric function: FL = MT/sen (PA), and belly length (BL), defined as the projection of the fascicle on the MTU plane, was obtained as BL = FL cos(PA) [18, 19]. Muscle belly shortening was calculated as the point-by-point difference between the projection of the fascicle on the MTU plane and that at rest. Calculated as such, the longitudinal displacement of the GM muscle belly is considered to represent the combined elongation of the distal aponeurosis, muscle, and free tendon [19].

For the tendon measurement, the position of the muscle tendon junction of gastrocnemius medialis (MTJ_GM_) was manually tracked (Tracker 6.1.3). Tendon elongation was calculated as the point-by-point difference between the MTJ_GM_ position and that at rest [20].

Muscle-tendon stiffness (k_M_) and tendon stiffness (k_T_) were calculated as the slope of the force-displacement curve in different force intervals [20]: 0–20%, 20–40%, 40–60%, 60–80%, 80–100% of maximum torque; the mean values of k_M_ and k_T_ (over all force intervals) were calculated as well.

After the MVCs, participants were asked to perform a series of maximal fix-end (isometric) explosive contractions at different torque levels (low, medium and high) to determine the rate of torque development (RTD: the first derivative of the torque-time signal) [11]. Based on these data the maximum RTD (RTD_peak_) reached during the explosive contractions was obtained [11].

Further details on data collection and analysis are reported in the supplementary materials.

### Statistical analysis

Statistical analysis was performed using Jamovi (v2.4.11). A Shapiro-Wilks test was used to assess the normality of the data. Participants’ characteristics were summarized using descriptive statistical methods. An unpaired t-test was applied to assess differences between groups in demographic, anthropometric and biochemical data. Both groups had moderate physical activity levels, yet marginally significant differences in IPAQ scores were observed. Hence, a one-way ANCOVA (with IPAQ score as a covariate) was used to determine possible differences in selected mechanical parameters. A two-way repeated measure ANCOVA (with the factors Group and Condition and the covariate IPAQ score) was performed to investigate differences in terms of k_M_, k_T_. Only the main effects of Group and the Group x Condition interaction are reported. Bonferroni correction was considered in the post hoc test. To assess the impact of differences in mechanical parameters between T2D and healthy controls, the effect size (i.e. partial eta-squared) was calculated to determine post hoc power. Linear regressions (with IPAQ score as a covariate) were used to test the relationships between parameters. A significance level of 0.05 was used for all statistical tests.

## Results

### Participants characteristics

The demographic, physical characteristics and biochemical profile of controls and T2D patients are reported in Table 1. The two cohorts were matched for age and BMI. No differences were also observed in terms of cognitive function (MMSE scores > 24 indicate no cognitive dysfunction) and functional capacity (IADL and ADL scores). IPAQ scores were marginally higher in healthy controls, but all participants could be considered moderately active (an IPAQ score < 700 MET-min week^− 1^ indicates a sedentary lifestyle, and an IPAQ score > 2500 indicates physically active participants).

Patients’ disease duration ranged from 1 to 20 years, and glycated hemoglobin ranged from 38 to 72 mmol molHb^− 1^ (5.6–8.7%). As expected, HbA1c and fasting glucose were higher in T2D patients. No differences were observed in AGE and RAGE (both in serum and skin biopsies) between groups.

### Muscle-tendon evaluation

**Fig. 1:**
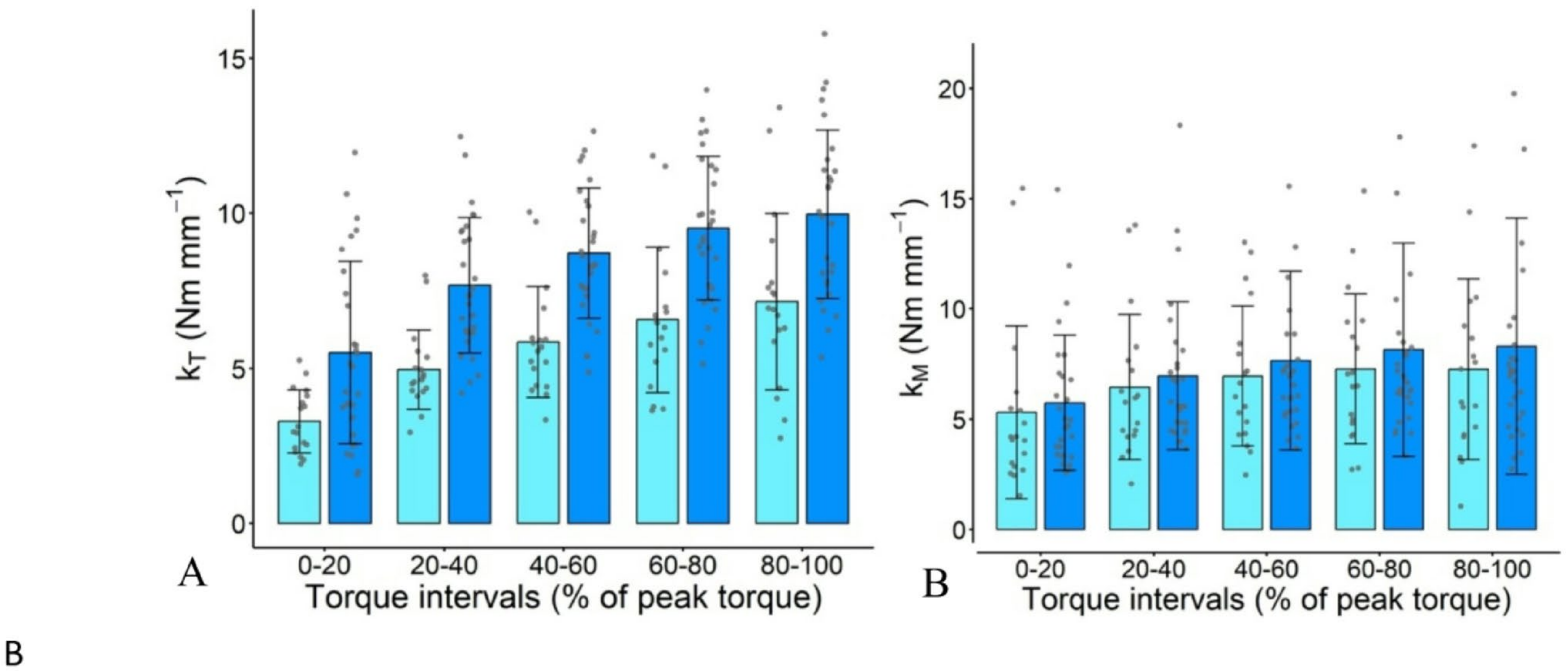
Tendon stiffness (k_T_, panel A) and muscle-tendon stiffness (k_M_, panel B) at different torque intervals. Data referring to controls are in light blue, to patients in blue. Individual values (dots) are reported along with means and standard deviations

Figure 1A and B show the values of tendon (k_T_) and muscle-tendon (k_M_) stiffness, respectively, in the five investigated intervals; the average values of k_T_ and k_M_, of peak torque and of maximum tissue displacement (AT elongation or muscle-tendon shortening) during the maximum voluntary contractions (MVCs) are reported in Table 2.

**Table 2:** Muscle-tendon evaluation data. Data are means ± SD. * *p* < 0.05: ** *p* < 0.001

		T2D	CR
*Maximal isometric contractions*			
Tendon stiffness evaluation	Peak torque (Nm)	95.8 ± 33.4	78.9 ± 25.7
	Tendon elongation (mm)	13.7 ± 3.2	16.7 ± 4.9 *
	Average k_T_ (Nm mm^− 1^)	8.3 ± 2.0	5.6 ± 1.6 **
Muscle-tendon stiffness evaluation	Peak torque (Nm)	94.2 ± 30.1	87.3 ± 26.2
	Muscle shortening (mm)	16.5 ± 6.8	17.1 ± 8.0
	Average k_M_ (Nm mm^− 1^)	7.4 ± 3.8	6.6 ± 2.9
*Explosive contractions*			
Low	Peak torque (Nm)	33.0 ± 15.5	29.3 ± 18.3
Medium		59.1 ± 20.6	48.4 ± 19.8
High		91.9 ± 29.3	77.1 ± 25.7
Low	RTD_peak_ (Nm s^− 1^)	309 ± 169	268 ± 152
Medium		451 ± 208	352 ± 143
High		556 ± 220	473 ± 161

The values of peak torque attained during the evaluation of k_T_ were similar in patients and controls (ANCOVA group effect: *P* = 0.100), but tendon elongation was significantly reduced in patients (*P* = 0.011, effect size = 0.142, post hoc power = 0.769) (Table 2). In addition, patients showed higher values of k_T_ in all the investigated torque intervals (ANCOVA main effect: *P* < 0.001; interaction: *P* = 0.967) (Fig. 1A). For the mean k_T_, the difference between controls and T2D patients reached a post hoc power of 0.998 (effect size = 0.348).

No differences in k_M_ (ANCOVA main effect: *P* = 0.148; interaction: *P* = 0.705) or muscle-tendon shortening were observed between groups (Fig. 1B; Table 2), indicating that muscle function was maintained in T2D patients. Although patients, in the explosive contractions, tend to show higher values of peak torque and RTD_peak_ at all intensities, these differences never reach a significant level (Table 2).

**Fig. 2:**
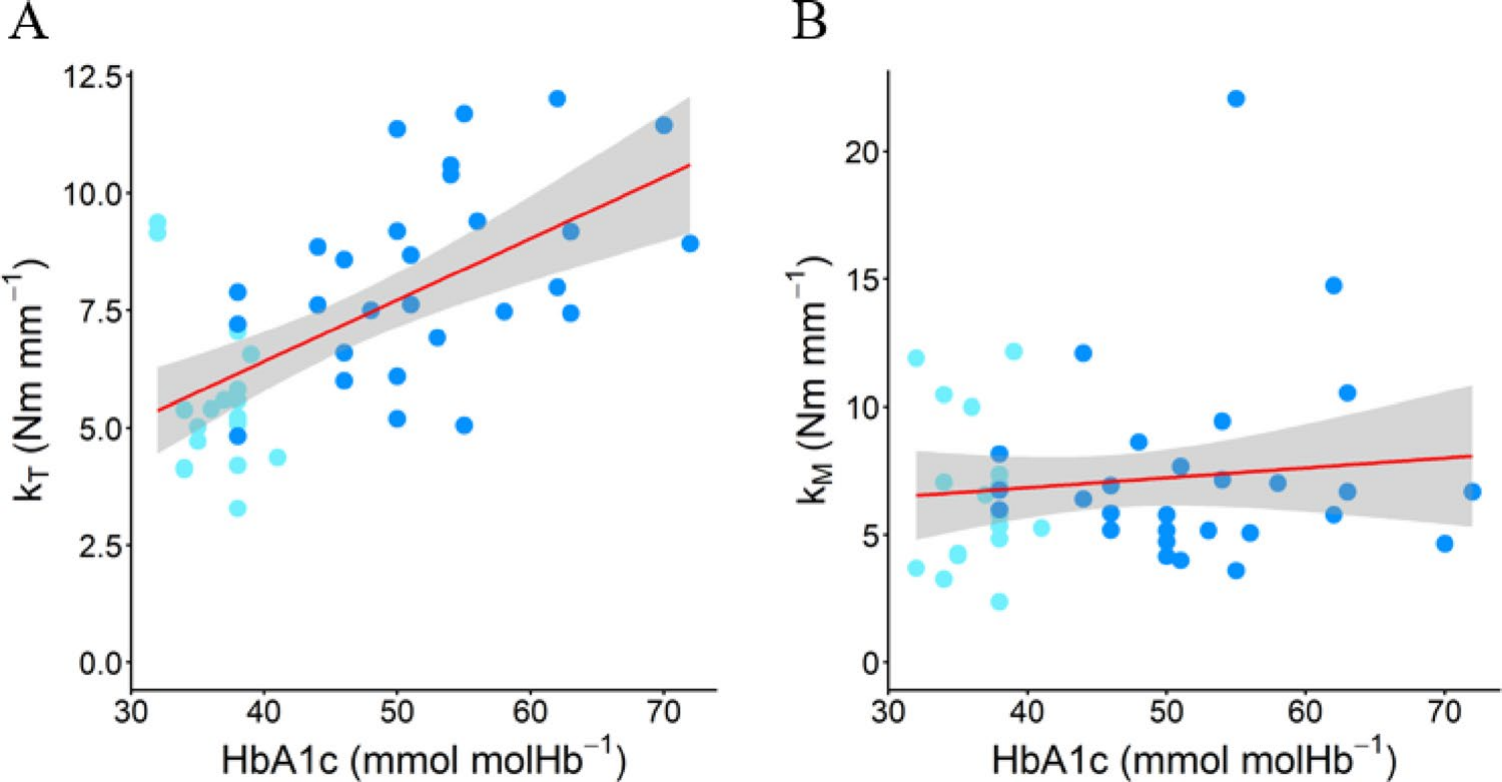
Correlations between tendon (**A**) and muscle-tendon (**B**) stiffness and HbA1c levels. Data are individual values (controls: light blue, patients: blue), and the grey-shaded area represents 95% of the confidence interval

Exploratory analyses were conducted to investigate possible correlations between mechanical (k_T_ or k_M_) and biochemical (HbA1c, AGE and RAGE) data. A positive correlation was observed between HbA1c and k_T_ (the average value at all torque intervals) (*r* = 0.610, *P* < 0.001, all participants) (Fig. 2), and this correlation persisted when analyzed within the T2D group (*r* = 0.438, *P* < 0.020). No correlation was observed between k_M_ (the average value at all torque intervals) and HbA1c levels, and no correlations were observed between k_T_ or k_M_ and AGE or RAGE values (as assessed either in serum or skin biopsies) or T2D duration (see supplementary materials).

No significant differences were observed in any of the parameters reported in Tables 1 and 2 when biopsy participants were compared to non-participants, in both groups (T2D patients and controls, see supplementary materials).

## Discussion

This study investigated possible alterations in muscle and tendon mechanics in physically active and controlled T2D patients, compared with a healthy, matched cohort. In addition, we investigated the possible association between muscle and tendon mechanical alterations, and the concentrations of non-enzymatic glycation products (HbA1c, AGE, and RAGE) in skin biopsies and serum.

Our data indicate that tendon stiffness is increased in T2D patients even when diabetes is controlled (and in the absence of peripheral neuropathies). On the contrary, muscle-tendon stiffness and the muscle’s mechanical output (e.g., explosive force capacity) appear to be unaffected.

Muscle and tendon stiffness are important functional parameters that strongly influence a muscle’s mechanical output. In the healthy population, an increase in muscle or tendon stiffness could be considered a useful adaptation since these parameters are well correlated with force transmission capacity (from contractile elements to joint motion), i.e. the capacity to exert force rapidly in response to different tasks (that can be investigated by assessing the rate of force development, RTD). Indeed, a direct correlation between patellar/Achilles tendon stiffness and RTD is consistently reported in the literature [21–23], and similar results were also observed for muscle stiffness [23, 24]. Tendon stiffness decreases with ageing [25] and inactivity [25], increases with training [26] and is increased in some pathological conditions (e.g. Duchenne’s dystrophy [27] and Parkinson’s disease [28]). In these pathologies, as well as in diabetes, an increase in tendon stiffness is generally the result of a decreased capacity of tendon elongation and an impairment in collagen quality, associated with a reduced recoil of elastic energy, which could constitute a mechanical disadvantage during daily locomotion tasks [9].

We observed, in agreement with previous studies, that people with T2D exhibit higher tendon stiffness (and reduced tendon elongation) as compared with a healthy cohort of the same age and with a similar level of physical activity. Couppè et al. (2016) [10] reported that Achilles tendon modulus (which represents tendon stiffness after accounting for tendon dimensions) is greater in diabetic patients (with controlled and poorly controlled diabetes) compared to age-matched controls (for age, BMI and IPAQ scores); while these authors observed no differences in tendon collagen cross-linking, they reported higher AGE content in the skin of patients as compared to controls.

We observed no differences in AGE and RAGE content in serum and skin between diabetic patients and controls, and found no association between AGE or RAGE content and muscle and tendon mechanics. Our findings agree with previous studies that indicate that AGE values are similar in diabetic patients without chronic diabetic complications and in controls [13], unlike the markedly increased rate observed in complication-prone patients [29]. As indicated by Zellers et al. (2021) [13], collagen disorganization, rather than AGEs content, is associated with the tendon mechanical proprieties; the lack of relationship between AGE content and tendon mechanical proprieties (observed in Zeller’s study as well as in Patel et al. (2024) [30]) challenges the long-standing assumption that AGE accumulation translates to altered tendon mechanics. Among the other factors that could be held responsible for increased tendon stiffness is the general state of inflammation, a feature of Achilles tendinopathy [31] that may contribute to the development of tendon complications in these individuals. In addition, as suggested by Wells-Knecht et al. (1197) [32], oxidative stress is not always increased in the extracellular milieu in diabetes, and this may also explain the lack of differences in AGE/RAGE content between diabetic patients and controls observed in this study.

As indicated in the introduction, recent studies indicate an association between tendon mechanical parameters and another glycation end product, glycated haemoglobin [7, 8]. Accordingly, in this study, we observed a significant correlation between HbA1c levels and tendon stiffness (the slope of the torque-tendon elongation relationship); a significant correlation between HbA1c and tendon modulus (the slope of the tendon stress-strain curve) was also reported by [30].

Training interventions that increase tendon elongation are thus expected to decrease tendon stiffness in diabetic patients, thereby improving their locomotor capacity; this was recently observed and reported by Magris et al. (2024) [33].

Only few studies investigated the effects of diabetes on muscle stiffness. AGEs accumulation on skeletal muscle in people with T2D could potentially induce muscle atrophy and dysfunction [34, 35], leading to diabetic myopathy. Fang et al. (2024) [36] reported lower values of active stiffness (but equal values of passive stiffness) in gastrocnemius medialis (assessed by means of shear wave elastography) in diabetic patients compared to controls and suggested that the decline in muscle function could affect the patient’s ability to perform daily activities. In this study, muscle stiffness was calculated accounting for the contribution of elastic tissues proximal to the tendon (muscle-tendon stiffness) and was unaffected by T2D. However, since the tendons and aponeuroses cannot be considered mechanically in series, our data likely reflect the force-length characteristics of the aponeurosis and suggest that muscle belly displacement is unaffected in individuals with controlled diabetes. Regular physical activity could mitigate AGE accumulation in soft tissues [37]. Hence, the physical activity level of our T2D patients (although only moderate) and the use of antidiabetic medications to control the pathology could have prevented AGE accumulation in the muscles, thus preserving their mechanical characteristics.

It would be interesting to understand whether increased tendon stiffness in the absence of functional deficit may predispose to other alterations, such as future tendinopathy or reduced adaptability under stress. However, data reported in this cross-sectional study and available literature do not allow us to answer this question. Prospective studies of adequate duration are required to verify this hypothesis.

### Limitations

The results of this study should be considered specific for well-controlled T2D patients. Indeed, patients with uncontrolled diabetes may exhibit larger alterations at the muscle-tendon level.

The level of physical activity was quantified by means of the IPAQ questionnaire, and then categorised according to the cut-offs proposed in the literature. All participants could be classified as moderately active, despite a mild difference in physical activity levels between the two cohorts. Patients with higher or lower levels of physical activity may exhibit different responses.

Although an association between tendon mechanical parameters and glycated haemoglobin has also been reported in other studies [7, 8, 30], the suggestion that HbA1c could act as a non-invasive biomarker of altered tendon mechanics remains hypothetical, given the cross-sectional design and the limited sample size of our study. Longitudinal studies with larger sample sizes are required to verify this hypothesis.

As pointed out by [30], care should be taken when considering the effects of polypharmacy on tendon properties; as an example, statin use [38], and high cholesterol levels [39] have been associated with tendon complications, while metformin can improve tendon structure, reducing the risk of tendon diseases [40].

## Conclusions

The hyperglycaemic environment that leads to increased HbA1c levels stiffens of the Achilles tendon, even in controlled T2D patients with a moderate physically active lifestyle; in these patients, functional abilities (e.g., rapid force production) could be maintained despite the increase in tendon stiffness. Although this study is cross-sectional and has a limited sample size, our data suggest a potential role of HbA1c as a non-invasive biomarker of altered tendon mechanics in people with diabetes.

## Supplementary Information

Below is the link to the electronic supplementary material.

Supplementary file1 (DOCX 51 KB)

## Data Availability

The data supporting this study’s findings are available from the corresponding author upon reasonable request.

## References

[1] Lapolla A, Traldi P, Fedele D (2001) AGE in micro- and macroangiopathy. Contrib Nephrol 10–21. 10.1159/000060063 10.1159/000060063 11125555

[2] Jørgensen ME, Kristensen JK, Husted GR et al (2016) The Danish Adult Diabetes Registry. CLEP 8:429–434. 10.2147/CLEP.S99518 10.2147/CLEP.S99518 PMC5098513 27843339

[3] Lee W-S, Kim J (2017) Diabetic cardiomyopathy: where we are and where we are going. Korean J Intern Med 32:404–421. 10.3904/kjim.2016.208 28415836 10.3904/kjim.2016.208 PMC5432803

[4] Akturk M, Karaahmetoglu S, Kacar M, Muftuoglu O (2002) Thickness of the supraspinatus and biceps tendons in diabetic patients. Diabetes Care 25:408. 10.2337/diacare.25.2.408 11815529 10.2337/diacare.25.2.408

[5] Nichols AEC, Muscat SN, Loiselle AE (2021) Short-Duration RAGE Antagonism Transiently Disrupts Tendon Homeostasis and does not Alter Diabetic Tendon Healing. 2021.05.11.443619

[6] Almurdhi MM, Reeves ND, Bowling FL et al (2016) Reduced Lower-Limb Muscle Strength and Volume in Patients With Type 2 Diabetes in Relation to Neuropathy, Intramuscular Fat, and Vitamin D Levels. Diabetes Care 39:441–447. 10.2337/dc15-0995 26740641 10.2337/dc15-0995 PMC5317239

[7] Monte A, Vilimek D, Uchytil J et al (2025) High levels of glycated haemoglobin (HbA1c) are associated with lower knee joint cartilage quality and higher knee joint symptoms in healthy individuals. Eur J Appl Physiol 125:885–894. 10.1007/s00421-024-05646-5 39482452 10.1007/s00421-024-05646-5

[8] Magris R, Uchytil J, Cipryan L et al (2025) Elevated glycated haemoglobin affects Achilles tendon properties and walking capacity in healthy people without a diagnosis of diabetes. Sci Rep 15:16077. 10.1038/s41598-025-01219-4 40341657 10.1038/s41598-025-01219-4 PMC12062501

[9] Petrovic M, Maganaris CN, Deschamps K et al (2018) Altered Achilles tendon function during walking in people with diabetic neuropathy: implications for metabolic energy saving. J Appl Physiol (1985) 124:1333–1340. 10.1152/japplphysiol.00290.2017 29420151 10.1152/japplphysiol.00290.2017

[10] Couppé C, Svensson RB, Kongsgaard M et al (2016) Human Achilles tendon glycation and function in diabetes. J Appl Physiol 120:130–137. 10.1152/japplphysiol.00547.2015 26542519 10.1152/japplphysiol.00547.2015

[11] Maffiuletti NA, Aagaard P, Blazevich AJ et al (2016) Rate of force development: physiological and methodological considerations. Eur J Appl Physiol 116:1091–1116. 10.1007/s00421-016-3346-6 26941023 10.1007/s00421-016-3346-6 PMC4875063

[12] Grant WP, Sullivan R, Sonenshine DE et al (1997) Electron microscopic investigation of the effects of diabetes mellitus on the Achilles tendon. J Foot Ankle Surg 36:272–278 discussion 330. 10.1016/s1067-2516(97)80072-5 9298442 10.1016/s1067-2516(97)80072-5

[13] Zellers JA, Eekhoff JD, Walk RE et al (2021) Human Achilles tendon mechanical behavior is more strongly related to collagen disorganization than advanced glycation end-products content. Sci Rep 11:24147. 10.1038/s41598-021-03574-4 34921194 10.1038/s41598-021-03574-4 PMC8683434

[14] Lawton MP, Brody EM (1969) Assessment of Older People: Self-Maintaining and Instrumental Activities of Daily Living. Gerontologist 9:179–186 5349366

[15] Craig CL, Marshall AL, Sjöström M et al (2003) International Physical Activity Questionnaire: 12-Country Reliability and Validity: Medicine &. Sci Sports Exerc 35:1381–1395. 10.1249/01.MSS.0000078924.61453.FB 10.1249/01.MSS.0000078924.61453.FB 12900694

[16] Folstein MF, Folstein SE, McHugh PR (1975) Mini-mental state. A practical method for grading the cognitive state of patients for the clinician. J Psychiatr Res 12:189–198. 10.1016/0022-3956(75)90026-6 1202204 10.1016/0022-3956(75)90026-6

[17] Farris DJ, Lichtwark GA (2016) UltraTrack: Software for semi-automated tracking of muscle fascicles in sequences of B-mode ultrasound images. Comput Methods Programs Biomed 128:111–118. 10.1016/j.cmpb.2016.02.016 27040836 10.1016/j.cmpb.2016.02.016

[18] Monte A, Tecchio P, Nardello F et al (2022) Influence of muscle-belly and tendon gearing on the energy cost of human walking. Scandinavian Med Sci Sports 32:844–855. 10.1111/sms.14142 10.1111/sms.14142 PMC9304283 35138687

[19] Wakeling JM, Blake OM, Wong I et al (2011) Movement mechanics as a determinate of muscle structure, recruitment and coordination. Phil Trans R Soc B 366:1554–1564. 10.1098/rstb.2010.0294 21502126 10.1098/rstb.2010.0294 PMC3130442

[20] Maganaris CN, Paul JP (2002) Tensile properties of the in vivo human gastrocnemius tendon. J Biomech 35:1639–1646. 10.1016/S0021-9290(02)00240-3 12445617 10.1016/s0021-9290(02)00240-3

[21] Bojsen-Møller J, Magnusson SP, Rasmussen LR et al (2005) Muscle performance during maximal isometric and dynamic contractions is influenced by the stiffness of the tendinous structures. J Appl Physiol (1985) 99:986–994. 10.1152/japplphysiol.01305.2004 15860680 10.1152/japplphysiol.01305.2004

[22] Massey GJ, Balshaw TG, Maden-Wilkinson TM et al (2017) The influence of patellar tendon and muscle-tendon unit stiffness on quadriceps explosive strength in man. Exp Physiol 102:448–461. 10.1113/EP086190 28205264 10.1113/EP086190

[23] Monte A, Zignoli A (2021) Muscle and tendon stiffness and belly gearing positively correlate with rate of torque development during explosive fixed end contractions. J Biomech 114:110110. 10.1016/j.jbiomech.2020.110110 33302182 10.1016/j.jbiomech.2020.110110

[24] Kubo K, Ikebukuro T (2019) Changes in joint, muscle, and tendon stiffness following repeated hopping exercise. Physiol Rep 7. 10.14814/phy2.14237 10.14814/phy2.14237 PMC6789417 31605467

[25] Magnusson SP, Kjaer M (2019) The impact of loading, unloading, ageing and injury on the human tendon. J Physiol 597:1283–1298. 10.1113/JP275450 29920664 10.1113/JP275450 PMC6395417

[26] Bohm S, Mersmann F, Arampatzis A (2015) Human tendon adaptation in response to mechanical loading: a systematic review and meta-analysis of exercise intervention studies on healthy adults. Sports Med - Open 1:7. 10.1186/s40798-015-0009-9 27747846 10.1186/s40798-015-0009-9 PMC4532714

[27] Lacourpaille L, Hug F, Guével A et al (2015) Non-invasive assessment of muscle stiffness in patients with duchenne muscular dystrophy. Muscle Nerve 51:284–286. 10.1002/mus.24445 25187068 10.1002/mus.24445

[28] Monte A, Magris R, Nardello F et al (2023) Muscle shape changes in Parkinson’s disease impair function during rapid contractions. Acta Physiol 238:e13957. 10.1111/apha.13957 10.1111/apha.13957 36876976

[29] Yu Y, Thorpe SR, Jenkins AJ et al (2006) Advanced glycation end-products and methionine sulphoxide in skin collagen of patients with type 1 diabetes. Diabetologia 49:2488–2498. 10.1007/s00125-006-0355-8 16955213 10.1007/s00125-006-0355-8

[30] Patel SH, Campbell NWC, Emenim CE et al (2024) Patellar tendon biomechanical and morphologic properties and their relationship to serum clinical variables in persons with prediabetes and type 2 diabetes. J Orthop Res 42:1653–1669. 10.1002/jor.25816 38400550 10.1002/jor.25816 PMC11222058

[31] Dakin SG, Newton J, Martinez FO et al (2018) Chronic inflammation is a feature of Achilles tendinopathy and rupture. Br J Sports Med 52:359–367. 10.1136/bjsports-2017-098161 29118051 10.1136/bjsports-2017-098161 PMC5867427

[32] Wells-Knecht MC, Lyons TJ, McCance DR et al (1997) Age-dependent increase in ortho-tyrosine and methionine sulfoxide in human skin collagen is not accelerated in diabetes. Evidence against a generalized increase in oxidative stress in diabetes. J Clin Invest 100:839–846. 10.1172/JCI119599 9259583 10.1172/JCI119599 PMC508256

[33] Magris R, Monte A, Nardello F et al (2024) Effects of minute oscillation stretching training on muscle and tendon stiffness and walking capability in people with type 2 diabetes. Eur J Appl Physiol. 10.1007/s00421-024-05596-y 39249539 10.1007/s00421-024-05596-y PMC11746953

[34] Snow LM, Thompson LV (2009) Influence of Insulin and Muscle Fiber Type in Nε-(Carboxymethyl)-Lysine Accumulation in Soleus Muscle of Rats with Streptozotocin-Induced Diabetes Mellitus. Pathobiology 76:227–234. 10.1159/000228898 19816082 10.1159/000228898 PMC2835375

[35] Chiu C-Y, Yang R-S, Sheu M-L et al (2016) Advanced glycation end-products induce skeletal muscle atrophy and dysfunction in diabetic mice via a RAGE-mediated, AMPK-down-regulated, Akt pathway. J Pathol 238:470–482. 10.1002/path.4674 26586640 10.1002/path.4674

[36] Fang X, Han Z, Kang Y et al (2024) Preliminary Study on the Evaluation of Skeletal Muscle Damage in Patients with Diabetes Mellitus Using Ultrasonic Shear Wave Elastography. Altern Ther Health Med 30 37820656

[37] Couppé C, Svensson RB, Grosset J-F et al (2014) Life-long endurance running is associated with reduced glycation and mechanical stress in connective tissue. AGE 36:9665. 10.1007/s11357-014-9665-9 24997017 10.1007/s11357-014-9665-9 PMC4150896

[38] Deren ME, Klinge SA, Mukand NH, Mukand JA (2016) Tendinopathy and Tendon Rupture Associated with Statins. JBJS Rev 4:e4. 10.2106/JBJS.RVW.15.00072 27490216 10.2106/JBJS.RVW.15.00072

[39] Tilley BJ, Cook JL, Docking SI, Gaida JE (2015) Is higher serum cholesterol associated with altered tendon structure or tendon pain? A systematic review. Br J Sports Med 49:1504–1509. 10.1136/bjsports-2015-095100 26474596 10.1136/bjsports-2015-095100 PMC4680137

[40] Chang R, Tu T-Y, Hung Y-M et al (2022) Metformin use is associated with a lower risk of rotator cuff disease in patients with Type 2 diabetes mellitus. Diabetes Metab 48:101368. 10.1016/j.diabet.2022.101368 35760373 10.1016/j.diabet.2022.101368

