# Supplementary material for: Impact of controlled type 2 diabetes on muscle-tendon mechanics
Source: Acta Diabetol. 2026 May 6;63(7):1239–46. doi: 10.1007/s00592-026-02705-5 (PMC13395907; doi:10.1007/s00592-026-02705-5)
Supplement: Supplementary file 1 — Supplementary file1 (DOCX 51 KB) [file 592_2026_2705_MOESM1_ESM.docx]

**Impact of well-controlled type 2 diabetes on muscle-tendon mechanics**

SUPPLEMENTARY MATERIALS

*Participants (sample size)*

The sample size was calculated *a-priori* (G*Power 3.1.9.4) with an effect size of 0.345 (calculated based on tendon strain values at maximum force, reported by Couppé et al., 2016), an alfa-level of 0.05, a statistical power of 0.8, two groups of participants (T2D and controls (CR)), 5 measurements (time intervals) and a correlation coefficient between repeated measurements of 0.5. The total sample size was 42.

*Skin Biopsies*

Skin biopsies were performed in the volar surface of the upper arm, to have the least possible aesthetic impact, with a 4-mm biopsy punch under local anaesthetic (1% lidocaine). Two sutures and a band-aid dressing were placed in the biopsied skin area. Skin biopsies were immediately frozen in liquid nitrogen for subsequent AGE and RAGE quantification (Dunn et al. 1991).

*Measurements of AGE and RAGE*

Serum and tissue sample levels of RAGE and AGE were measured using enzyme-linked immunosorbent assays (ELISA) according to the manufacturer’s instructions: Human AGE ELISA Kit (FineTest, Cat. No. EH0622) and the Human RAGE ELISA Kit (BioVendor Group, Cat. No. RD191116200R).

For AGE determination, the provided calibration curve (0.0 ng/mL–20 ng/mL) was employed to calculate AGE concentrations. The intra-assay precision (within-run) was 5.12%, 5.89%, and 5.34% at 0.63 ng/mL, 2.41 ng/mL, and 10.26 ng/mL, respectively. Similarly, the inter-assay precision (between-run) was 5.23%, 4.88%, and 4.98% at 0.6 ng/mL, 2.47 ng/mL, and 9.96 ng/mL, respectively. For RAGE determination, the provided calibration curve (0.0 pg/mL–3.2 pg/mL) was used to calculate RAGE concentrations. The intra-assay precision (within-run), expressed as the coefficient of variation (CV%), was 2.6% and 5.3% at 1.595 ng/mL and 1.075 ng/mL, respectively, while the inter-assay precision (between-run) was 5.5% and 8.8% at 1.644 ng/mL and 4.742 ng/mL, respectively.

*Evaluation of muscle and tendon stiffness*

*Data collection*

A standardized warm-up based on 30 unloaded calf raises was performed before the experimental protocol. After that, participants were accommodated on a dynamometer (Cybex NORM, Lumex Inc., Ronkonkoma, New York, USA) with a trunk and pelvis strap; the dominant foot was fixed to the dynamometer footplate with the knee fully extended (i.e. 0°) and the hip flexed at 85°. Participants performed 10 sub-maximal voluntary contractions (i.e. familiarization session) during which the ankle axis of rotation, defined as the imaginary line connecting medial and lateral malleoli, was carefully aligned to the dynamometer axis of rotation (Arampatzis et al. 2004). The ankle angle was set to 0 degrees of plantarflexion, accounting for the soft tissue compression that occurs at low torque levels to avoid angular changes during contractions (Fukunaga et al. 1996).

The torque-shortening relationship of medial gastrocnemius medialis muscle belly and the torque-elongation curve of the Achille’s tendon were obtained by combining torque and ultrasound measurements during a series of maximal voluntary contractions (MVCs). The participants were instructed to increase the ankle joint moment gradually (for 1-2 s) until their maximum and then to maintain it for 2-3 s (Maganaris and Paul, 2002); visual feedback of the torque values was provided to better graduate the torque increase. Participants performed at least six MVCs (3+3 contractions for the evaluation of MTU and tendon stiffness, respectively) with 2 min of recovery in between (Monte et al., 2021). During each MVC, participants received strong verbal encouragement.

An ultrasound apparatus, with a 6-cm linear array probe operating at 60 Hz, was utilized (Telemed Mycrus Ext-1, Lituania) to record muscle architecture behaviour and the Achilles tendon displacement. For the torque-shortening curve of the muscle belly, three MVCs were performed with the probe placed in the sagittal plane on the most prominent part of the GM muscle belly to obtain data regarding muscle thickness and pennation angle during contraction (Hauraix et al., 2015). For the torque-elongation curve of the Achille’s tendon, three MVCs were performed with the same probe placed along the middle-longitudinal axis of the muscle-tendon unit at the GM muscle-tendon junction to obtain the tendon elongation (Magnusson et al., 2001).

A soft gel pad was interposed between the probe and the skin to reduce muscle compression and its negative effects (Monte, 2021) and an elastic bandage was utilized to minimize the probe movement relative to the skin. To ensure the best image quality, the position of the scanning probe was corrected until the muscle or the muscle-tendon unit (MTJ) and surrounding connective tissue were clearly visible (Van Hooren et al., 2020); the depth and width of the ultrasound recordings were adjusted to maintain constant the sample rate.

Torque data were acquired at 1000 Hz with a PowerLab System (PowerLab, ADInstrument) using the ad hoc software (LabChart v8.1.13, ADIstrument). At the beginning of the ultrasound video acquisition, a 3V square wave trigger was activated to synchronize ultrasound data with the torque signal.

*Data analysis*

For the MVCs and the explosive contractions, the ankle moment generated by the plantar-flexor muscles was corrected for the possible DC offset imposed by leg position (Bakenecker et al. 2019). Net ankle torque was then filtered with a zero-phase fourth- order low pass (25 Hz) Butterworth filter.

In addition, we did not collect and subtract the possible contribution of the antagonist muscles on the net ankle torque.

For the muscle-belly ultrasound measurements, a customised version of a semi-automatic tracking algorithm was used to determine muscle thickness (MT) and pennation angle (PA) of four fascicles frame by frame (Farris and Lichtwark, 2016). At the end of the auto-tracking, every frame of the tracked parameters was visually examined to check the algorithm’s accuracy. Whenever MT or PA was deemed inaccurate, the points were manually repositioned. MT was defined as the distance between the aponeuroses perpendicular to the midline of the muscle, whereas PA was defined as the angle between the fascicles and the deep aponeurosis (average of the four fascicles) (Seynnes et al., 2015). Fascicle length (FL) was then calculated using a trigonometric function: FL = MT/sen (PA). The belly length (BL) was defined as the projection of the fascicle on the MTU plane and was obtained as BL = FL cos (PA) (Wakeling et al., 2011; Monte et al., 2022).

For the tendon elongation measurement, the position of the MTJ_GM_ was tracked manually (Tracker 6.1.3). Tendon elongation was calculated as the point-by-point difference between the MTJ_GM_ position and that at rest. All ultrasound data were filtered with a second-order low pass (5 Hz) Butterworth filter.

Muscle-tendon stiffness (k_M_) and tendon stiffness (k_T_) were calculated based on the torque-longitudinal displacement data of the GM muscle belly e of the MTJ_GM_, respectively. In turn, the longitudinal displacement of the GM muscle belly was considered to represent the combined elongation of the distal aponeurosis, muscle, and free tendon.

To reconstruct the torque-displacement curves, torque data (acquired at a frequency of 100 Hz) were sub-sampled at the ultrasound sampling rate (60 Hz). The curves (each trial for each subject) were fitted with a second-order polynomial function forced to through zero (Hannah and Folland, 2015). In further analysis, only trials with an R^2^ of the fitted curve higher than 0.9 were considered. Finally, k_M_ e k_T_ were calculated as the slope of the force-displacement curve in different force intervals (Maganaris and Paul, 2002): 0-20%, 20-40%, 40-60%, 60-80%, 80-100% of maximum torque; the mean values of k_M_ and k_T_ over all force intervals were calculated as well. The values of MTJ_GM_ displacement, MTU_GM_ displacement and peak torque during MVCs were averaged across the three trials of each subject.

After the MVCs, the participants were asked to perform nine maximal fix-end explosive contractions (1 s of duration and 1 min of recovery in between) at different torque levels (low, medium and high). During these explosive contractions, participants were instructed to push as fast as possible, avoiding any countermovement.

The rate of torque displacement (RTD) was calculated as the first derivative of the torque-time signal (Maffiuletti et al., 2016). Torque onset was identified manually (visually), as suggested by (Tillin et al., 2010, 2012) and peak torque and peak RTD (RTD_peak_) were identified in the explosive contractions at all intensities (low, medium, high).

For the MVCs and the explosive contractions, the ankle moment generated by the plantar-flexor muscles was corrected for the gravitational moment (determined during a passive joint rotation driven by the dynamometer) (Bakenecker et al., 2019); net ankle torque was then filtered with a second order low pass (25 Hz) Butterworth filter. The offline analyses were conducted using customized Matlab scripts (R2022b).

*Results of the linear regressions (r; p-value) between mechanical parameters (i.e. kT and kM) and disease duration or biochemical data (HbA1c, AGE and RAGE).*

|  | k_T_ | k_M_ |
| --- | --- | --- |
| Disease duration (years) | 0.223; 0.434 | 0.502; 0.164 |
| Hb1Ac (mmol molHb^-1^) | 0.610; <0.001 | 0.286; 0.169 |
| AGE serum (µg mL^-1^) | 0.174; 0.713 | 0.333; 0.074 |
| AGE skin (µg mL^-1^) | 0.292; 0.156 | 0.221; 0.716 |
| RAGE serum (ng mL^-1^) | 0.206; 0.415 | 0.234; 0.507 |
| RAGE skin (ng mL^-1^) | 0.217; 0.390 | 0.213; 0.920 |

*Participants' characteristic and muscle-tendon characteristics in those who accepted the skin biopsy and those who denied their consent*.

P-value refers to unpaired t-test or U-Mann Whitney test (if the normality assumption was not met).

|  | CR (with biopsy) | CR (without biopsy) | | p | | T2D (with biopsy) | | T2D (without biopsy) | | p | |  |
| --- | --- | --- | --- | --- | --- | --- | --- | --- | --- | --- | --- | --- |
| n (M/F) | 11 (7/4) | 7 (3/4) | |  | | 24 (16/8) | | 4 (3/1) | |  | |  |
| Age (years) | 64.27 ± 4.86 | 63.29 ± 6.21 | | 0,75 | | 61.88 ± 4.41 | | 66.50 ± 3.00 | | 0,055 | |  |
| Body mass (kg) | 75.14 ± 13.24 | 69.71 ± 9.55 | | 0,364 | | 79.58 ± 9.72 | | 81.00 ± 17.91 | | 0,813 | |  |
| Stature (m) | 1.70 ± 0.12 | 1.67 ± 0.05 | | 0,554 | | 1.72 ± 0.08 | | 1.74 ± 0.10 | | 0,683 | |  |
| BMI (kg m^-2^) | 25.97 ± 2.34 | 25.09 ± 3.45 | | 0,525 | | 26.93 ± 2.53 | | 26.52 ± 3.11 | | 0,775 | |  |
| Disease onset (years) | - | - | | - | | 7.90 ± 7.16 | | 10.67 ± 8.33 | | 0,394 | |  |
| Hb1Ac 3y (mmol molHb^-1^) | - | - | | - | | 52.42 ± 9.36 | | 52.25 ± 5.32 | | 0,973 | |  |
| Hb1Ac (mmol molHb^-1^) | 35.82 ± 2.56 | 37.29 ± 2.29 | | 0,236 | | 51.03 ± 6.44 | | 49.05 ± 6.98 | | 0,973 | |  |
| Hb1Ac (%) | 5.43 ± 0.23 | 5.56 ± 0.21 | | 0,236 | | 6.95 ± 0.86 | | 6.93 ± 0.49 | | 0,577 | |  |
| Glucose (mmol L^-1^) | 5.18 ± 0.47 | 4.89 ± 0.34 | | 0,167 | | 7.06 ± 1.68 | | 7.85 ± 0.99 | | 0,199 | |  |
| Creatinine (µmol L^-1^) | 78.6 ± 13.2 | 72.4 ± 10.9 | | 0,318 | | 77.2 ± 18.0 | | 79.2 ± 14.1 | | 0,828 | |  |
| Total Cholesterol (mmol L^-1^) | 5.13 ± 1.23 | 5.49 ± 0.71 | | 0,497 | | 3.86 ± 0.77 | | 3.88 ± 0.46 | | 0,97 | |  |
| HDL cholesterol (mmol L^-1^) | 1.14 ± 0.58 | 1.00 ± 0.38 | | 0,267 | | 1.29 ± 0.39 | | 1.55 ± 0.59 | | 0,873 | |  |
| Triglycerides (mmol L^-1^) | 1.55 ± 0.45 | 1.79 ± 0.40 | | 0,86 | | 1.26 ± 0.29 | | 1.28 ± 0.29 | | 0,264 | |  |
| AGE serum (µg mL^-1^) | 0.28 ± 0.13 | 0.28 ± 0.17 | | 0,536 | | 0.56 ± 1.57 | | 0.23 ± 0.04 | | 0,590 | |  |
| AGE skin (µg mL^-1^) | 0.04 ± 0.02 | - | | - | | 0.03 ± 0.01 | | - | | - | |  |
| RAGE serum (ng mL^-1^) | 0.69 ± 0.17 | 0.71 ± 0.19 | | 0,838 | | 1.17 ± 1.53 | | 0.68 ± 0.19 | | 0,590 | |  |
| RAGE skin (ng mL^-1^) | 0.11 ± 0.14 | - | | - | | 0.13 ± 0.07 | | - | | - | |  |
| ADL (score) | 6.0 ± 0.0 | 6.0 ± 0.0 | | 1 | | 6.0 ± 0.0 | | 6.0 ± 0.0 | | 1 | |  |
| IADL (score) | 8.0 ± 0.0 | 8.0 ± 0.1 | | 1 | | 8.0 ± 0.0 | | 8.0 ± 0.0 | | 1 | |  |
| IPAQ (MET-min week^-1^) | 1698 ± 593 | 1436 ± 538 | | 0,359 | | 1187 ± 326 | | 982 ± 140 | | 0,232 | |  |
| MMSE (score) | 28.7 ± 1.1 | 29.1 ± 1.0 | | 0,442 | | 29.1 ± 1.1 | | 27.8 ± 1.0 | | 0,033 | |  |
| Peak torque (Nm) | 80.51 ± 30.59 | 76.32 ± 17.35 | | 0,748 | | 98.85 ± 34.27 | | 77.34 ± 22.19 | | 0,240 | |  |
| Tendon elongation (mm) | 15.99 ± 4.93 | 17.89 ± 5.00 | | 0,438 | | 13.78 ± 3.15 | | 13.23 ± 3.94 | | 0,757 | |  |
| Average k_T_ (Nm mm^-1^) | 5.89 ± 1.98 | 5.04 ± 0.62 | | 0,293 | | 8.52 ± 2.00 | | 6.80 ± 1.54 | | 0,115 | |  |
| Peak torque (Nm) | 88.25 ± 31.43 | 85.79 ± 17.01 | | 0,858 | | 95.68 ± 30.89 | | 85.14 ± 26.90 | | 0,527 | |  |
| Muscle shortening (mm) | 18.41 ± 9.95 | 15.57 ± 3.07 | | 0,479 | | 16.25 ± 7.16 | | 17.74 ± 4.19 | | 0,692 | |  |
| Average k_M_ (Nm mm^-1^) | 7.03 ± 3.63 | 6.05 ± 1.23 | | 0,504 | | 7.69 ± 4.00 | | 5.41 ± 1.43 | | 0,262 | |  |
| Peak torque (Nm) low | 24.35 ± 12.78 | 35.67 ± 23.14 | | 0,232 | | 32.99 ± 16.34 | | 33.19 ± 10.94 | | 0,982 | |  |
| Peak torque (Nm) medium | 44.30 ± 18.83 | 53.74 ± 21.18 | | 0,362 | | 59.09 ± 21.17 | | 59.02 ± 19.24 | | 0,995 | |  |
| Peak torque (Nm) high | 72.74 ± 28.44 | 84.06 ± 20.90 | | 0,379 | | 91.09 ± 29.96 | | 97.29 ± 28.54 | | 0,703 | |  |
| RTDpeak (Nm s^-1^) low | 221.7 ± 86.9 | 328.8 ± 199.6 | | 0,168 | | 301.4 ± 171.4 | | 352.0 ± 174.2 | | 0,590 | |  |
| RTDpeak (Nm s^-1^) medium | 337.4 ± 136.2 | 371.7 ± 160.1 | | 0,650 | | 443.7 ± 214.3 | | 494.3 ± 187.3 | | 0,465 | |  |
| RTDpeak (Nm s^-1^) high | 437.6 ± 176.8 | | 527.8 ± 125.9 | | 0,259 | | 537.5 ± 232.9 | | 665.3 ± 51.6 | | 0,322 | |
